# Thoracic vascular injury remains the leading cause of death in traumatic haemorrhage: Analysis of injury patterns and time to death

**DOI:** 10.1007/s00068-026-03131-6

**Published:** 2026-03-02

**Authors:** Marcus Wannberg, Hannah Lindén, Mahmood Ul Hassan, Poya Ghorbani, Lovisa Strömmer, Carl-Magnus Wahlgren

**Affiliations:** 1 Department of Molecular Medicine and Surgery, Vascular Surgery, Karolinska Institutet, Stockholm, Sweden; 2 Division of Biostatistics, Institute of Environmental Medicine, Karolinska Institutet, Stockholm, Sweden; 3 Division of Surgery and Oncology, Department of Clinical Science, Intervention and Technology, Karolinska Institute, Stockholm, 17177 Sweden; 4 Department of Vascular Surgery, Karolinska University Hospital, Stockholm, Sweden

**Keywords:** Haemorrhage, Blunt trauma, Penetrating trauma, Vascular injury, Death

## Abstract

**Purpose:**

Understanding injury patterns and temporal dynamics of traumatic haemorrhagic death is essential for developing targeted interventions. This study characterized anatomic bleeding locations, specific vascular injuries, and survival times in patients who died from traumatic haemorrhage.

**Methods:**

Retrospective single centre study of trauma patients who died in hospital from traumatic haemorrhage during index admission between 2007 and 2023. Data were extracted from systematic mortality reviews, trauma registries, medical records, and autopsy reports. Survival times were analyzed in relation to bleeding location and specific vascular injuries.

**Results:**

In the overall cohort (*n* = 226), the median age was 33 years (IQR 23–51); male patients 85% (191/226). Penetrating trauma (58%, 130/226) dominated over blunt trauma (42%, 96/226) (*p* = 0.013). Thorax (*n* = 94, 42%) was the most common region for haemorrhage death followed by multiple locations (*n* = 89, 39%), abdomen (*n* = 33, 15%), extremities (*n* = 6, 2.7%), and neck (*n* = 4, 1.8%). Time-to-death categorization revealed that most deaths (40%) occurred between 60 and 120 min after injury, with 21% dying within the first 60 min. Thoracic fatal haemorrhage caused the shortest survival times (median 77 min, IQR 55–197), while abdominal haemorrhage had the longest survival time (127 min, IQR 93–251). The survival probability after 90 min for abdominal bleeding region was OR 3.89, 95% CI 1.27–11.9, *p* = 0.018. Thoracic aorta was the most frequent (42%, 47/113) identified vascular injury with the shortest survival (75 min, IQR 55–189).

**Conclusions:**

Thoracic haemorrhage, particularly when involving major vessels, represented the most lethal bleeding source with the shortest survival duration; in contrast, abdominal haemorrhage was associated with a comparatively longer survival window. These findings emphasize the critical importance of minimizing time to definitive haemorrhage control through expeditious surgical management strategies.

**Supplementary Information:**

The online version contains supplementary material available at 10.1007/s00068-026-03131-6.

## Background

Traumatic haemorrhage remains the leading cause of potentially preventable death following injury, accounting for up to 30–40% of trauma-related mortality worldwide in both civilian and military populations [1, 2]. Despite advances in trauma care, approximately one-fourth of trauma deaths could be prevented with timely intervention, as nearly half of haemorrhagic fatalities occur within the first two hours after injury [2]. Thoracic vascular trauma is particularly lethal, with up to 71% of patients with major vessel injuries dying before reaching hospital care [3]. Injury patterns vary by setting with penetrating trauma is more prevalent in military contexts while both blunt and penetrating mechanisms are common in civilian trauma. However, the central challenge remains with rapid identification and control of haemorrhage, especially from non-compressible torso location [4]. 

Annual mortality among patients in haemorrhagic shock has remained relatively unchanged over time [5]. Survival is strongly influenced by the time between injury and definitive haemorrhage controls [6, 7]. In urban trauma systems, 74% of hemorrhagic deaths occur in the prehospital setting or within one hour of hospital arrival [6]. 

A more comprehensive understanding of early haemorrhagic death is essential for developing targeted interventions, particularly for managing non-compressible torso haemorrhage (NCTH). The objective of this study is to further characterize injury patterns and time to death in trauma haemorrhage mortality.

## Methods

### Study population

This was a retrospective single centre study at a university trauma center including 226 patients with death from traumatic haemorrhage between January 1st, 2007, and December 31st, 2023. The total number of trauma admissions and deaths during the study period was 23,896 and 2335, respectively (Table 1). The study was approved by the national ethical review authority (DNR 2024-05997-02).

**Table 1:** Annual distribution of trauma admissions, 30-day mortality and haemorrhage death

Year	Trauma admissions	30-day mortality	Haemorrhage deaths
2007	1350	69 (5.1%)	12
2008	1597	71 (4.4%)	10
2009	1510	60 (4.0%)	5
2010	1463	71 (4.9%)	11
2011	1516	75 (5.0%)	12
2012	1506	80 (5.3%)	10
2013	1539	126 (8.2%)	10
2014	1514	197 (13%)	14
2015	1409	197 (14%)	24
2016	1409	155 (11%)	7
2017	1491	239 (16%)	21
2018	1393	295 (21%)	18
2019	1245	199 (16%)	19
2020	1377	179 (13%)	16
2021	1345	134 (10%)	8
2022	1153	127 (11%)	11
2023	1079	163 (15%)	18

### Inclusion and exclusion study criteria

All trauma patients who arrived at the trauma centre and subsequently died in hospital due to traumatic haemorrhage during the index admission were included. Patients declared dead at the scene and not transported to hospital were not included. Classification of bleeding region and vascular injury was based on structured multidisciplinary mortality review and available clinical and forensic information medical record review and, when available, autopsy reports. Resuscitative interventions may have been initiated in some patients who arrived without signs of life, based on initial prehospital information and local protocols. Final case classification was determined retrospectively during multidisciplinary mortality review using all available information.

### Study methodology

All trauma deaths were subjected to systematic mortality review at our institution by a multidisciplinary peer review committee (MPRC) as previously described [8]. The MPRC conducted structured analysis of all trauma-related deaths to identify patterns of care, cause of death and potential preventability. All patients who died from traumatic haemorrhage during the index admission were identified through this systematic mortality review process. Registry data for these patients were extracted from the local and the national Swedish Trauma Registry (SweTrau) [9]. The anatomical regions of haemorrhage and major vascular injuries were extracted from MPRC protocols, medical records and forensic autopsy reports.

### Definitions

Haemorrhage was classified into five major anatomic regions: neck, thorax, abdomen, extremities, and multiple locations. Multiple locations were used if there were two or more major bleeding locations.

Variables were defined according to the Utstein trauma template for uniform reporting of data following major trauma [10]. This standardized framework guided the categorization of anatomic bleeding regions (thorax, abdomen, neck, extremities, or multiple regions) and injury mechanisms (traffic-related, penetrating, blunt, and falls). Emergency trauma intervention was defined as the first key emergency intervention performed for treatment and stabilization of the patient including damage control thoracotomy and laparotomy. Survival time was defined as time from alarm until time of death in hospital pronounced by physician in charge.

### Statistical analysis

Data was presented as median with interquartile range (IQR) and odds ratio (OR) with 95% confidence interval (CI). Descriptive statistics were performed for patient characteristics and outcomes. Differences in patient characteristics across outcome variable were assessed using Pearson’s Chi-squared test, Fisher’s exact test, or the Kruskal-Wallis test, as appropriate, depending on variable type and distribution.

Ordinal regression models were used to analyze “survival time”, an outcome variable representing the time from injury to death, categorized as ≤ 60 min, 61–120 min, 121–240 min, 241–360 min, and > 360 min. The primary explanatory variable was “bleeding region”, classified as: thorax, abdomen, extremities, and multiple locations. Model coefficients were estimated using maximum likelihood and presented with 95% confidence intervals and *p*-values. Model performance was evaluated using Akaike Information Criterion (AIC), Bayesian Information Criterion (BIC), and root mean squared error (RMSE).

To assess the temporal trend in mortality, a simple linear regression was performed with the number of deaths per year as the dependent variable and calendar year as the independent variable.

All statistical tests were two-sided, p-values < 0.05 was considered significant. All the statistical analyses were performed with R v4.5.1 (R Core Team, 2025).

## Results

### Patient characteristics traumatic haemorrhage deaths

**Fig. 1:**
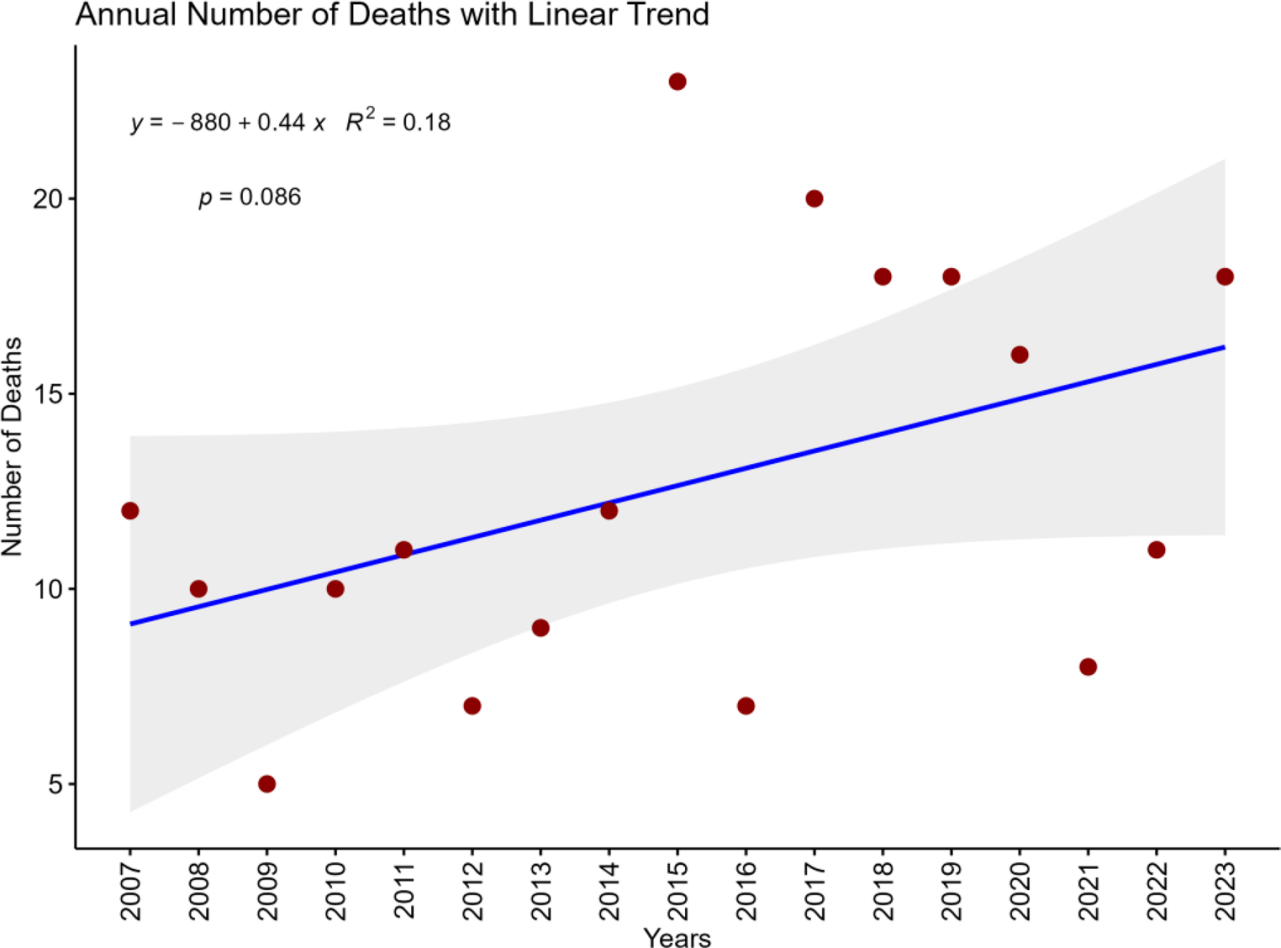
The annual numbers of traumatic haemorrhage deaths from 2007 to 2023 (overall cohort *n* = 226)

In the overall cohort (*n* = 226), the median age was 33 years (IQR 23–51), with a predominance of male patients (85%, 191/226) (Table 2). Prehospital cardiac arrest data were available for 161/226 patients (71%). Among those, 131/161 (81%) had documented cardiac arrest prior to hospital arrival. There was an increasing non-significant trend of traumatic haemorrhage deaths during the study period (Fig. 1). The annual variation of haemorrhage death related to sex is showed in supplementary Fig. 1. During the study period, annual trauma admissions decreased, and a higher overall mortality was registered (Table 1). Penetrating trauma (58%, 130/226) dominated over blunt trauma (42%, 96/226) (*p* = 0.013). More specifically stabbed by knife 26% (*n* = 58) was most common, followed by shot by firearms 23% (*n* = 52), high-energy fall 19% (*n* = 44), motor vehicle collision 12% (*n* = 27), and motorcycle collision 7.5% (*n* = 17). Prehospital cardiac arrest was documented in 81% of patients (131/161). The overall median Injury Severity Score (ISS) was 50 (IQR 30–75) and median New Injury Severity Score (NISS) was 57 (IQR 41–75). The initial base excess and international normalized ratio (INR) were − 15 (IQR − 22.4 - -7.4) and 1.3 (IQR 1.1–1.6), respectively.

**Table 2:** Patient and injury characteristics by major anatomic bleeding region

Characteristics	Thorax *N* = 94	Abdomen *N* = 33	Neck *N* = 4	Extremities *N* = 6	Multiple regions *N* = 89	*P*-value^1^
Age (IQR)	33 (23, 47)	32 (22, 55)	43 (23, 64)	47 (27, 64)	33 (24, 51)	0.8
Sex:						0.2
Female	13 (14%)	2 (6.1%)	0 (0%)	1 (17%)	19 (21%)	
Male	81 (86%)	31 (94%)	4 (100%)	5 (83%)	70 (79%)	
Type of Injury:						0.088
Blunt	33 (35%)	18 (55%)	0 (0%)	3 (50%)	42 (47%)	
Penetrating	61 (65%)	15 (45%)	4 (100%)	3 (50%)	47 (53%)	
Prehospital cardiac arrest	57/70 (81%)	13/18 (72%)	4/4 (100%)	4/5 (80%)	53/64 (83%)	0.8
ISS (IQR)	54 (29, 75)	35 (26, 45)	23 (17, 29)	34 (21, 45)	57 (38, 75)	< 0.001
NISS (IQR)	66 (43, 75)	43 (34, 57)	30 (22, 34)	50 (36, 50)	66 (41, 75)	< 0.001
Injury mechanism:						
Motor vehicle collision	11 (12%)	3 (9.1%)	0 (0%)	0 (0%)	13 (15%)	
Motorcycle collision	9 (9.6%)	2 (6.1%)	0 (0%)	0 (0%)	6 (6.7%)	
Bicycle collision	0 (0%)	2 (6.1%)	0 (0%)	0 (0%)	1 (1.1%)	
Traffic: pedestrian	2 (2.1%)	1 (3.0%)	0 (0%)	1 (17%)	1 (1.1%)	
Traffic: other	0 (0%)	0 (0%)	0 (0%)	0 (0%)	3 (3.4%)	
Shot by firearm	22 (23%)	10 (30%)	2 (50%)	0 (0%)	18 (20%)	
Stabbed by knife	33 (35%)	7 (21%)	2 (50%)	2 (33%)	14 (16%)	
Struck or hit by blunt object	3 (3.2%)	4 (12%)	0 (0%)	1 (17%)	4 (4.5%)	
Low energy fall	1 (1.1%)	0 (0%)	0 (0%)	0 (0%)	0 (0%)	
High energy fall	12 (13%)	3 (9.1%)	0 (0%)	2 (33%)	27 (30%)	
Blast injury	0 (0%)	0 (0%)	0 (0%)	0 (0%)	1 (1.1%)	
Other	1 (1.1%)	1 (3.0%)	0 (0%)	0 (0%)	1 (1.1%)	

Data are presented as median (IQR) for continuous variables and n (%) for categorical variables. ISS = Injury Severity Score; NISS = New Injury Severity Score. ^1^Kruskal-Wallis rank sum test; Fisher’s exact test

### Anatomic bleeding regions

**Fig. 2:**
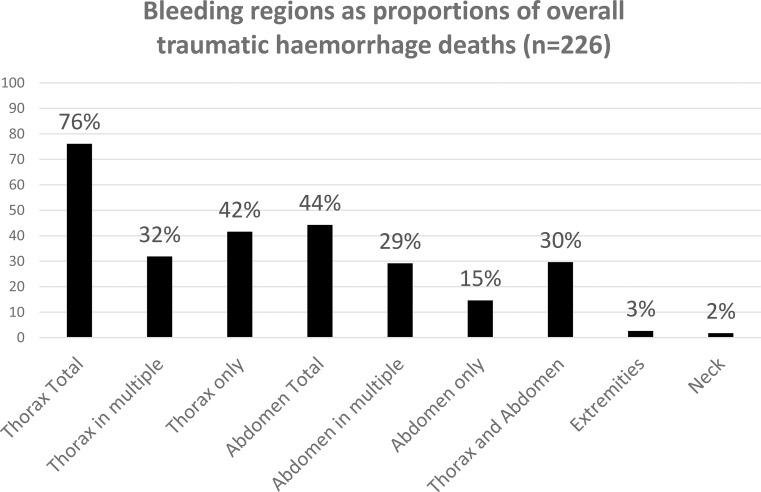
Bleeding regions, both localized to one region and combinations of regions, in traumatic haemorrhage deaths (non-disjunctive categories). *Bars show the proportion of all included cases with bleeding involved in each anatomical region (percentage of the total cohort); a single case may involve multiple bleeding regions*

Thorax (*n* = 94, 42%) was the most common region for haemorrhage death followed by multiple locations (*n* = 89, 39%), abdomen (*n* = 33, 15%), extremities (*n* = 6, 2.7%), and neck (*n* = 4, 1.8%) (Table 2). The multiple injury locations dominated by thorax+abdomen (*n* = 65; median age 32, IQR 24–50; 80% male; 57% blunt mechanism). In Fig. 2, all bleeding sites, both localized to one region and combinations of regions, are summarized. There was a significant difference observed in ISS between bleeding regions (*p* < 0.001), with multiple injury locations and thorax demonstrating the highest severity scores. The most common injury mechanism in thorax was stabbed by knife, in abdomen firearms, and for multiple injury locations high-energy falls. There were only penetrating mechanisms in the neck and an even distribution of both penetrating and blunt mechanisms in the extremities. Multinomial regression analysis did not show any relevant associations between bleeding region and injury mechanisms. The annual variation of haemorrhage death related to anatomic bleeding region is showed in supplementary Fig. 2.

### Bleeding region and time to hospital arrival and emergency trauma intervention

The median time from alarm to arrival at the scene was similar across anatomic bleeding regions (10 min, IQR 7–15). The overall median time from alarm to hospital arrival was 43 min (IQR 32–54), with no significant differences between bleeding regions (*p* = 0.5). Patients with penetrating trauma arrived at the hospital earlier than those with blunt trauma 44 (IQR 18) minutes vs. 50 (IQR 19) minutes (*p* = 0.013).

The overall median time from hospital arrival to initial emergency trauma intervention was 47 min (IQR 36–57). There were 107 thoracotomies and 52 laparotomies performed emergently; 44 were combined thoracotomy and laparotomy. Patients with extremity bleeding region experienced longer times to treatment 53 min (IQR 49–124) compared to other regions, although these differences did not reach statistical significance (*p* = 0.7).

### Bleeding region and survival time

**Fig. 3:**
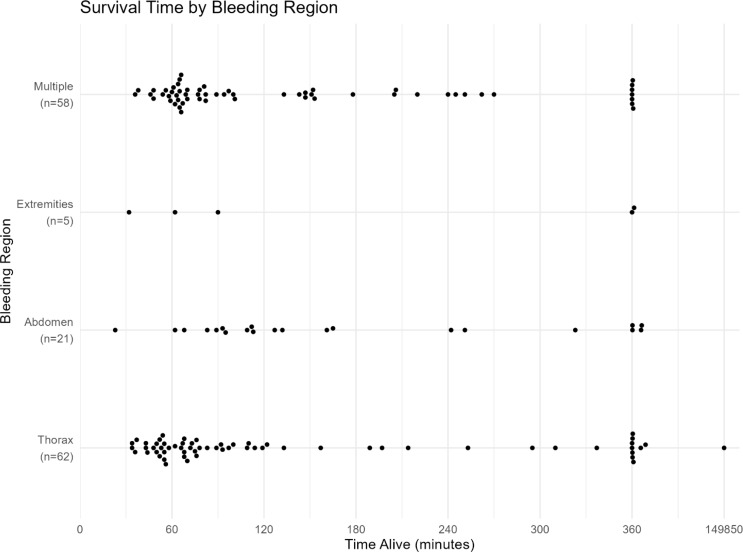
Survival time (minutes) and major bleeding region. Survival times beyond 360 min have been compressed in the graph allowing comparison of early survival patterns across major bleeding regions, while preserving the overall distribution of later times

When analyzing the time from injury to death, the median survival time varied between bleeding regions. Patients with fatal abdominal bleeding had the longest median survival time (127 min, IQR 93–251), followed by extremity bleeding (90 min, IQR 62–400), multiple site bleeding (82 min, IQR 64–205), and thoracic bleeding (77 min, IQR 55–197) (Fig. 3). The survival probability after 90 min (compared to ≤ 90 min) for abdominal bleeding region was OR 3.89, 95% CI 1.27–11.9, *p* = 0.018. The time data was not available for the four patients with fatal bleeding from the neck region.

Time-to-death categorization revealed that most deaths (40%) occurred between 60 and 120 min after injury, with 21% dying within the first 60 min (15% within 2–4 h). For thoracic bleeding, 31% of deaths occurred within 60 min after injury. There were few abdominal bleeding deaths recorded in the early time window and a higher risk after 90 min from injury (estimate = -0.15, SE = 0.057, df = 6, t = -2.66, *p* = 0.038).

### Specific vascular injuries and survival time

A specific vascular injury was identified in 113 fatal patients. The most frequent vessel injuries were thoracic aorta (*n* = 47, 41.6%), iliac vessels (*n* = 13, 11.5%), pulmonary vessels (*n* = 10, 8.8%), aortic arch/subclavian artery (*n* = 8, 7.1%), carotid artery (*n* = 6, 5.3%), inferior vena cava (*n* = 6, 5.3%), abdominal aorta (*n* = 6, 5.3%), and visceral vessels (*n* = 6, 5.3%).

There were observed differences in ISS between vascular injury groups (*p* = 0.026), where thoracic aortic injuries had the highest median ISS 75 (IQR 38–75). NISS values also differed significantly between vascular injury groups (*p* < 0.001), with thoracic aortic and pulmonary vessel injuries showing the highest median NISS (both 75).

For vascular injuries, the associated median time from hospital arrival to initial treatment varied considerably: pulmonary vessels 40 min (IQR 38–41), carotid artery 45 min (IQR 34–54), thoracic aorta 51 min (IQR 42–64), iliac vessels 54 min (IQR 28–67), visceral vessels 64 min (IQR 44–97), and femoral artery 96 min (IQR 67–124).

**Fig. 4:**
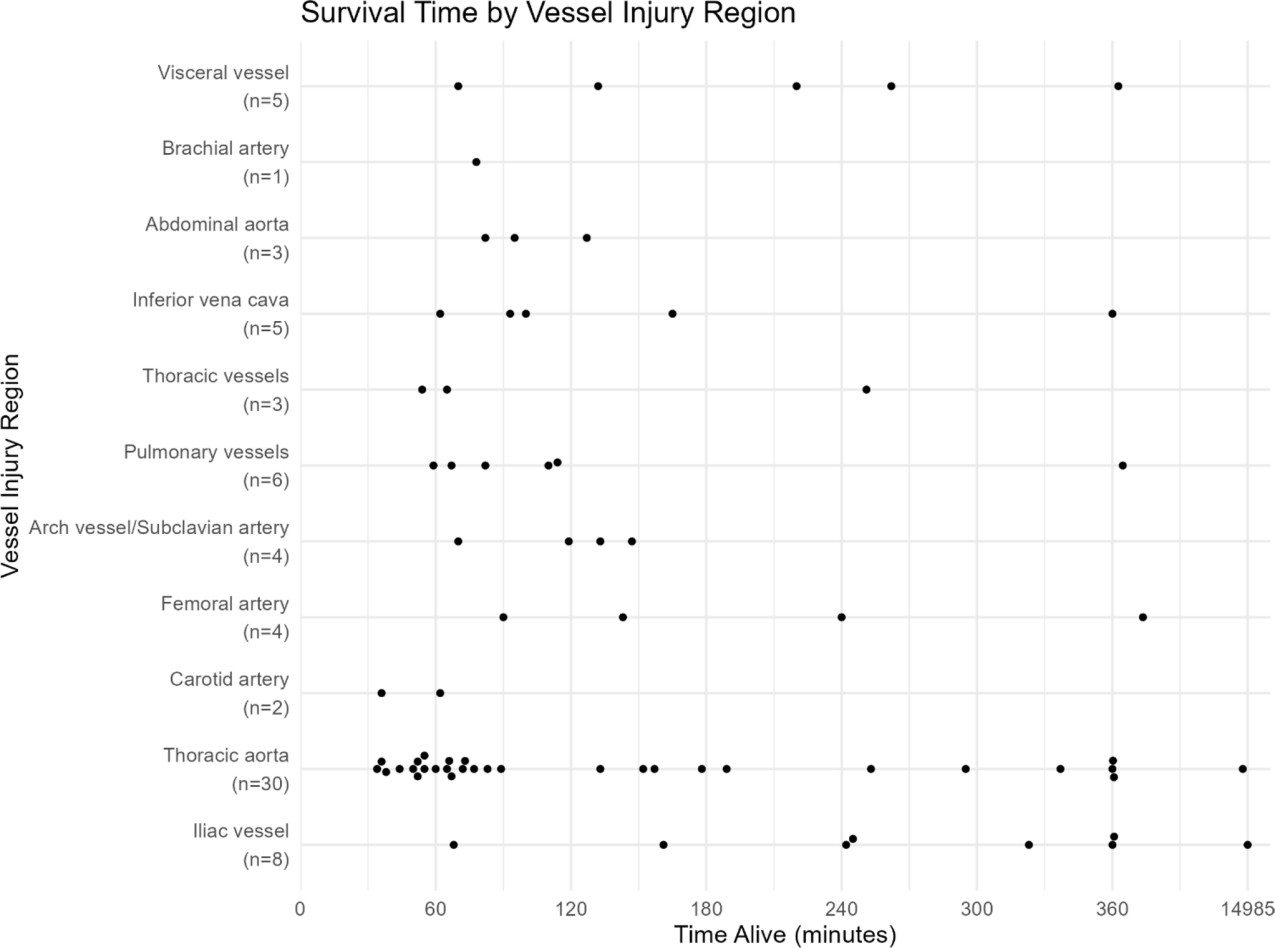
Survival time (minutes) and vessel injury region. Survival times beyond 360 min have been compressed in the graph allowing comparison of early survival patterns across vessel injury regions, while preserving the overall distribution of later times

Regarding associated survival time (Fig. 4), patients with iliac vessel injuries had the longest median survival time (284 min, IQR 202–466), followed by visceral vessel injuries (220 min, IQR 132–262) and femoral artery injuries (192 min, IQR 117–1952). Patients with thoracic aortic injuries had considerably shorter median survival time (75 min, IQR 55–189).

Time-to-death categorization for vascular injuries showed similar patterns as for the overall cohort, with the highest proportion of deaths (35%) occurring between 60 and 120 min after injury. However, thoracic aortic injuries showed a high proportion of early deaths, with 33% occurring within 60 min, while iliac vessel injuries had a high proportion of late deaths (38% occurring between 240 and 360 min and 38% after 360 min).

## Discussion

This study examines the characteristics and time patterns associated with traumatic haemorrhage deaths. These fatalities occurred predominantly in younger males. The chest was the most common site of fatal haemorrhage, particularly from penetrating trauma. Most deaths occurred within one to two hours of injury, with injuries to major chest vessel, especially the thoracic aorta, were associated with shorter survival times.

During the study period spanning the past two decades, substantial trauma-demographic changes have occurred. The number of firearm-related injuries has increased, and a regional reorganization has directed patients with severe trauma to designated trauma center, while less severely injured patients have been managed at other hospitals within the region. At the same time, an overall decline in trauma admissions has been observed in recent years. However, the annual numbers of traumatic haemorrhage deaths have been relatively stable with an increasing trend in recent years. Also, haemorrhage accounts for a relatively smaller proportion of the overall trauma-related deaths. Trauma registry reporting has improved, and a more structured multidisciplinary mortality review process has been implemented. Toward the end of the study period, prehospital physicians began pronouncing death at the scene of injury, with deceased patients subsequently transported directly for forensic autopsy. Patients with injuries incompatible with life or without response to initial resuscitation, in the context of anticipated prolonged transport time, were declared deceased at the scene. This may have had an impact on mortality outcome data at hospital level.

The chest or a combination of chest and abdominal injuries were the most frequently observed bleeding locations, so called NCTH. This aligns with previous findings indicating that isolated truncal haemorrhage accounts for approximately three-quarters of haemorrhagic deaths [6]. A small number of deaths were due to extremity haemorrhage, likely resulting from more proximal vascular injuries inaccessible to external compression. In a multicenter retrospective study, NCTH was distributed across the chest (25%), abdomen (41%), pelvis (31%), and unspecified regions (3%) [7]. That study included both survivors and non-survivors, which may explain the higher representation of abdominal bleeding sources.

The impact of time to death is a critical factor explored in this study. Most patients succumbed within 1–2 h post-injury, with thoracic bleeding sources being the most lethal. Blunt trauma involving multiple bleeding sites tended to result in slightly longer survival, and abdominal haemorrhage rarely caused death within the first hour. However, these findings should be interpreted with caution due to possible selection bias, as prehospital deaths were not included in the current study. In a comprehensive analysis of trauma-related haemorrhagic deaths within a metropolitan area over one year, the majority occurred either in the prehospital setting or within the first hour of hospital admission [6]. Previous studies report a median time to exsanguination of approximately two hours [7, 11]. 

In this urban setting, the overall median time from emergency alarm to hospital arrival was 43 min, followed by a median time of 47 min to initial emergency trauma intervention. Patients with penetrating injuries generally experienced shorter prehospital times. In a recent analysis of the UK Trauma Audit and Research Network National Data Registry, the median time to surgical intervention for patients in hemorrhagic shock was 3.1 h (IQR 1.6–12.2), including both survivors and non-survivors [5]. Trauma systems are continuously working to minimize the time to definitive haemorrhage control. These efforts include the prehospital administration of blood products and advanced resuscitative care, and implementing life-saving interventions earlier along the chain of survival [12–16]. 

In this study, chest haemorrhage involving major vascular injury was associated with the shortest survival times and remains particularly difficult to manage. However, fatal abdominal haemorrhage was associated with a comparatively longer survival window. Some patients who reach hospital with abdominal injuries involving major vessels, such as the abdominal aorta or inferior vena cava, most likely develop a retroperitoneal tamponade that provides a brief period of haemodynamic stability, allowing time for additional diagnostics to guide management. Effective management of these injuries, via open or endovascular techniques, is crucial and ideally performed in a hybrid operating suite [17]. 

This study has several limitations that must be acknowledged. Its retrospective design with inherent limitations including incomplete registry data, particularly regarding the exact time of death. Selection bias is possible, as patients who died at the scene were not included, potentially skewing data on survival times for specific bleeding sources and vessel injuries. Additionally, the precise contribution of each bleeding site to mortality may be difficult to assess. Nonetheless, autopsy and surgical reports were reviewed in detail to obtain the most accurate data possible. Furthermore, termination of resuscitation practices and documentation of prehospital CPR vary across systems and may influence observed time-to-death distributions, however, this is unlikely to affect classification of the anatomic bleeding source among included cases [18]. A 17-year study period is also likely to encompass changes in trauma management practice at both the pre-hospital and in-hospital levels.

In conclusion, this study further delineates injury patterns and time to death in patients who died from traumatic haemorrhage. Reducing the time to definitive haemorrhage control, including strategies of early use of prehospital blood products and expeditious surgical bleeding interventions, remains critical to further improve outcomes.

## Electronic supplementary material

Below is the link to the electronic supplementary material.

Supplementary Material 1

## Data Availability

Data available from the authors upon reasonable request.
