# Supplementary material for: Thoracic vascular injury remains the leading cause of death in traumatic haemorrhage: Analysis of injury patterns and time to death
Source: Eur J Trauma Emerg Surg. 2026 Mar 2;52(1):74. doi: 10.1007/s00068-026-03131-6 (PMC12953328; doi:10.1007/s00068-026-03131-6)

**Supplementary**

Figure 1. The annual distribution of traumatic haemorrhage death related to sex.


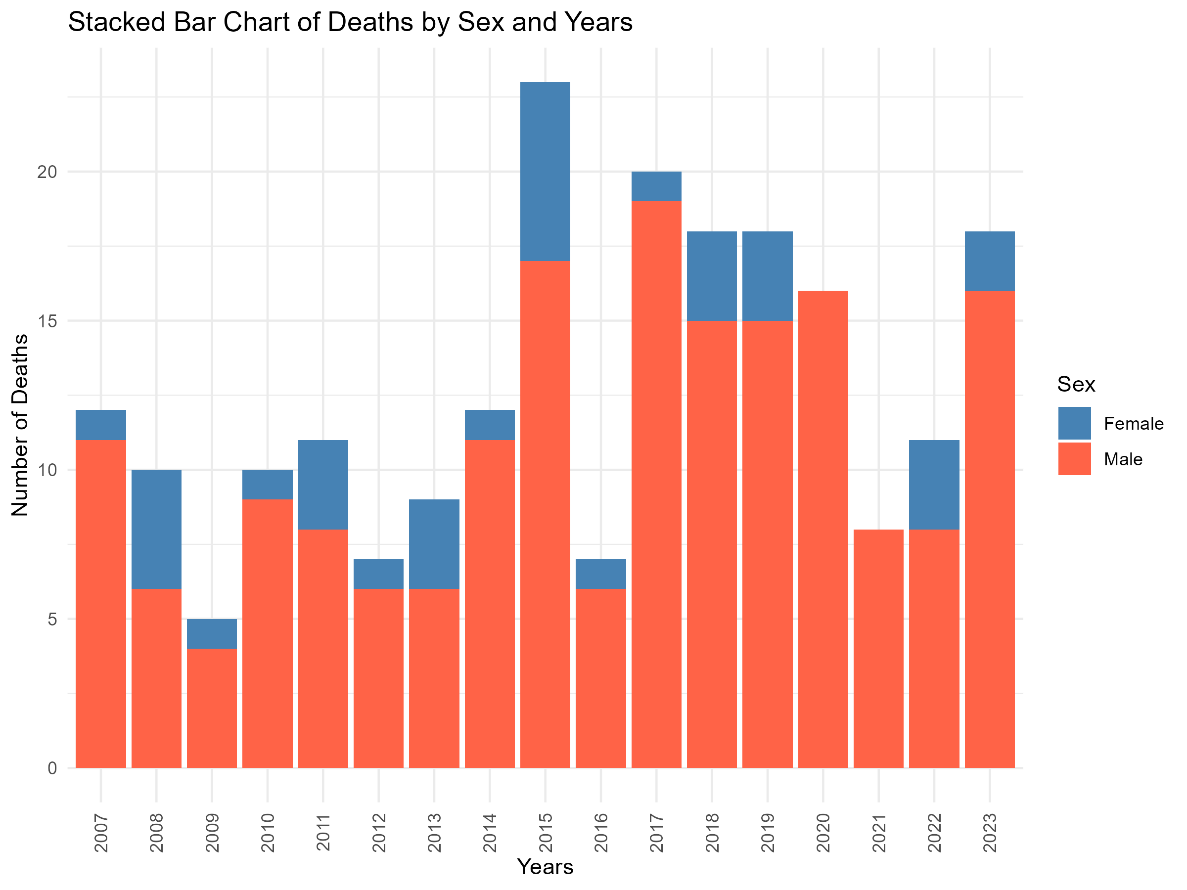


**Figure 2.** The annual distribution of traumatic haemorrhage death related to bleeding region.


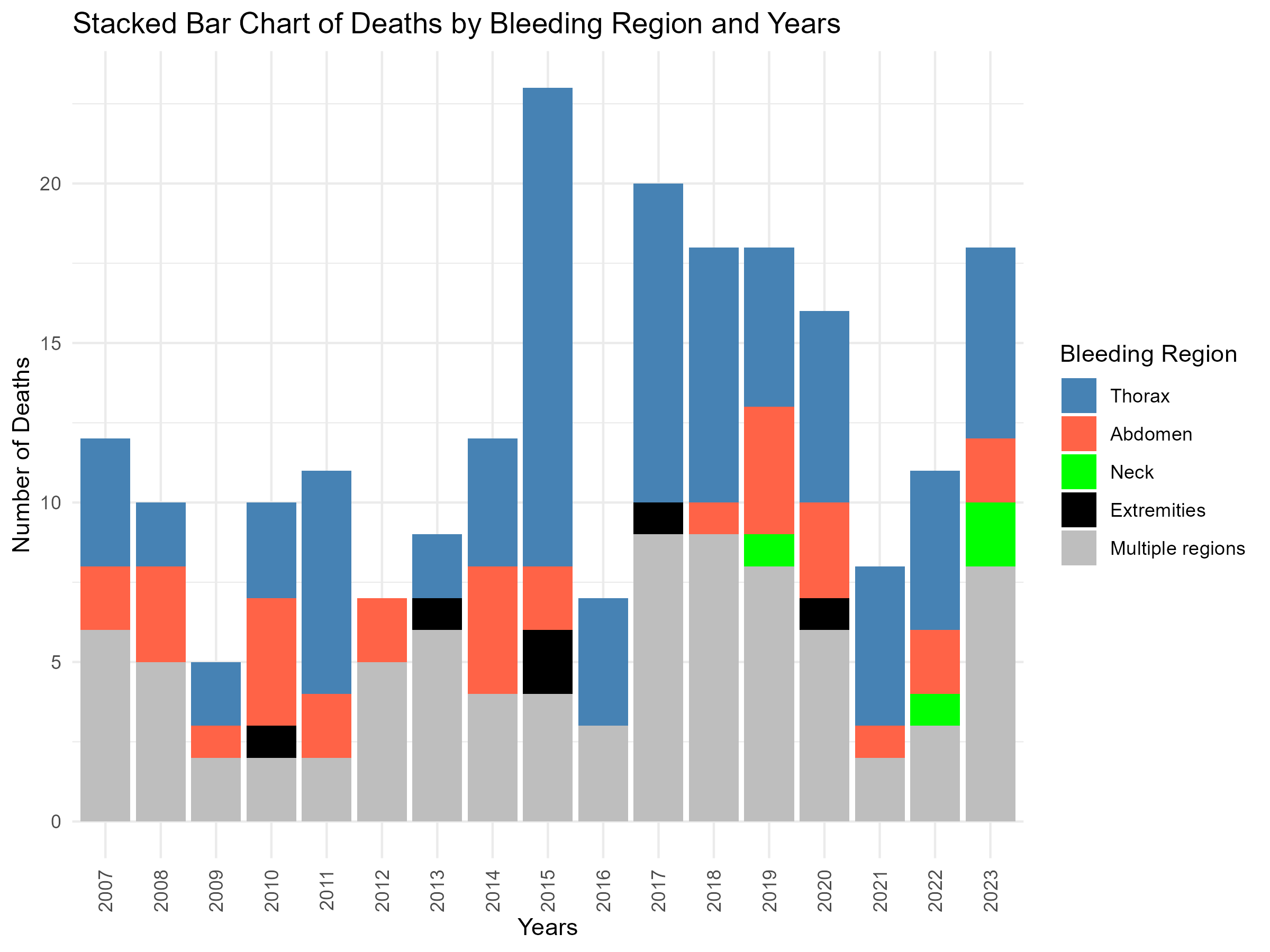

Supplement: Supplementary file 1 — Supplementary Material 1 [file 68_2026_3131_MOESM1_ESM.docx]
